# O11.6. WHO GETS IN TO EARLY PSYCHOSIS INTERVENTION SERVICES? A COMPARISON OF SERVICE USERS AND NON-USERS IN HEALTH ADMINISTRATIVE DATA

**DOI:** 10.1093/schbul/sby015.266

**Published:** 2018-04-01

**Authors:** Kelly Anderson, Ross Norman, Arlene MacDougall, Jordan Edwards, Lena Palaniyappan, Cindy Lau, Paul Kurdyak

**Affiliations:** 1 University of Western Ontario; 2 Western University; 3 Institute for Clinical Evaluative Sciences

## Abstract

**Background:**

There is a dearth of information on people with first-episode psychosis who do not access specialized early psychosis intervention (EPI) services. With this notable gap in knowledge comes the implicit assumption that nearly all cases of first-episode psychosis are detected and treated by EPI services. We sought to estimate the proportion of incident cases of non-affective psychosis who do not access these services, and to examine factors associated with EPI admission.

**Methods:**

Using health administrative data, we constructed a retrospective cohort of incident cases of non-affective psychosis in the catchment area of the Prevention and Early Intervention Program for Psychoses (PEPP) in London, Ontario between 1997 and 2013. This cohort was linked to primary data from PEPP to identify EPI-users. We used multivariate logistic regression to model socio-demographic and service factors associated with EPI admission.

**Results:**

Over 50% of suspected cases of non-affective psychosis did not have contact with the EPI program for screening or admission. Our findings suggest a clear gradient by age, with a decreasing likelihood of being treated in the EPI program with increasing age strata (age 46–50 years vs. age 16–20 years: OR=0.03, 95%CI=0.01–0.05). EPI-users are more likely to be male (OR=1.58, 95%CI=1.24–2.01), and less likely to live in areas of socioeconomic deprivation (OR=0.51, 95%CI=0.36–0.73). EPI-users also had a higher odds of psychiatrist involvement at the index diagnosis (OR=7.35, 95%CI=5.43–10.00), had a lower odds of receiving the index diagnosis in an outpatient setting (OR=0.50, 95%CI=0.38–0.65), and had a lower odds of prior alcohol-related (OR=0.42, 95%CI=0.28–0.63) and substance-related (OR=0.68, 95%CI=0.50–0.93) disorders.

**Discussion:**

Much of the prior research on EPI services is predicated on the belief that nearly all patients with first-episode psychosis are represented in these services, with little discussion or consideration of people who may be receiving care elsewhere in the health system. We need greater consideration of patients with first-episode psychosis who are not accessing EPI services – our findings suggest this group is sizable, and there may be socio-demographic and clinical disparities in access. Non-psychiatric health professionals could be targeted with interventions aimed at increasing detection and referral rates.

